# Ethyl 1-[2-(morpholin-4-yl)eth­yl]-2-[4-(trifluoro­meth­yl)phen­yl]-1*H*-benzimid­azole-5-carboxyl­ate

**DOI:** 10.1107/S1600536811014619

**Published:** 2011-04-29

**Authors:** Yeong Keng Yoon, Mohamed Ashraf Ali, Tan Soo Choon, Madhukar Hemamalini, Hoong-Kun Fun

**Affiliations:** a Institute for Research in Molecular Medicine, Universiti Sains Malaysia, Minden 11800, Penang, Malaysia; b X-ray Crystallography Unit, School of Physics, Universiti Sains Malaysia, 11800 USM, Penang, Malaysia

## Abstract

In the title compound, C_23_H_24_F_3_N_3_O_3_, the morpholine ring adopts a chair conformation. The benzimidazole ring is approximately planar, with a maximum deviation of 0.028 (1) Å for one of the unsubstituted C atoms. The benzimidazole ring makes dihedral angles of 35.66 (4) and 75.45 (5)° with the attached phenyl and morpholine rings, respectively. In the crystal structure, adjacent mol­ecules are linked *via* C—H⋯F and C—H⋯O hydrogen bonds to form a two-dimensional network.

## Related literature

For background to benzimidazoles, see: Boruah & Skibo (1994 ▶); Haugwitz (1982 ▶); Hisano (1982 ▶); Hubschwerlen (1992 ▶); Shi (1996 ▶). For ring conformations, see: Cremer & Pople (1975 ▶). For the stability of the temperature controller used in the data collection, see: Cosier & Glazer (1986 ▶).
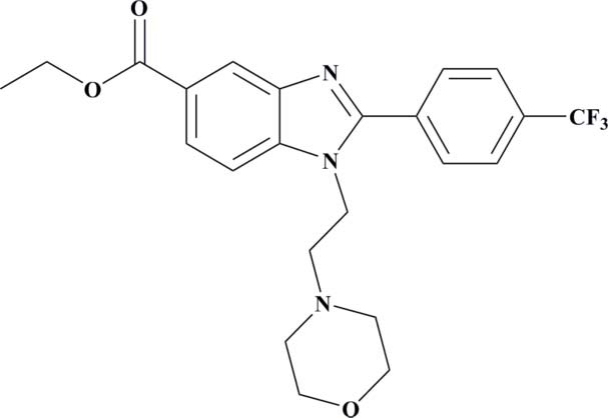


## Experimental

### 

#### Crystal data

C_23_H_24_F_3_N_3_O_3_
*M* *_r_* = 447.45
Triclinic, 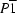

*a* = 10.1463 (2) Å
*b* = 10.5595 (2) Å
*c* = 11.5775 (2) Å
α = 96.868 (1)°
β = 109.638 (1)°
γ = 110.833 (1)°
*V* = 1050.83 (3) Å^3^
*Z* = 2
Mo *K*α radiation
μ = 0.11 mm^−1^
*T* = 100 K
0.51 × 0.33 × 0.19 mm

#### Data collection

Bruker SMART APEXII CCD diffractometer
Absorption correction: multi-scan (*SADABS*; Bruker, 2009 ▶) *T*_min_ = 0.945, *T*_max_ = 0.979
22546 measured reflections
6122 independent reflections
5266 reflections with *I* > 2σ(*I*)
*R*_int_ = 0.024

#### Refinement

*R*[*F* ^2^ > 2σ(*F* ^2^)] = 0.038
*wR*(*F* ^2^) = 0.106
*S* = 1.03
6122 reflections
290 parameters
H-atom parameters constrained
Δρ_max_ = 0.43 e Å^−3^
Δρ_min_ = −0.26 e Å^−3^

### 

Data collection: *APEX2* (Bruker, 2009 ▶); cell refinement: *SAINT* (Bruker, 2009 ▶); data reduction: *SAINT*; program(s) used to solve structure: *SHELXTL* (Sheldrick, 2008 ▶); program(s) used to refine structure: *SHELXTL*; molecular graphics: *SHELXTL*; software used to prepare material for publication: *SHELXTL* and *PLATON* (Spek, 2009 ▶).

## Supplementary Material

Crystal structure: contains datablocks global, I. DOI: 10.1107/S1600536811014619/hb5849sup1.cif

Structure factors: contains datablocks I. DOI: 10.1107/S1600536811014619/hb5849Isup2.hkl

Additional supplementary materials:  crystallographic information; 3D view; checkCIF report

## Figures and Tables

**Table 1:** Hydrogen-bond geometry (Å, °)

*D*—H⋯*A*	*D*—H	H⋯*A*	*D*⋯*A*	*D*—H⋯*A*
C2—H2*A*⋯F1^i^	0.95	2.51	3.4617 (15)	175
C10—H10*A*⋯O3^ii^	0.95	2.38	3.1889 (14)	143
C20—H20*A*⋯O2^iii^	0.99	2.52	3.4878 (14)	166

Symmetry codes: (i) 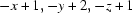; (ii) 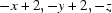; (iii) 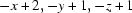.

**Fig. 1.**
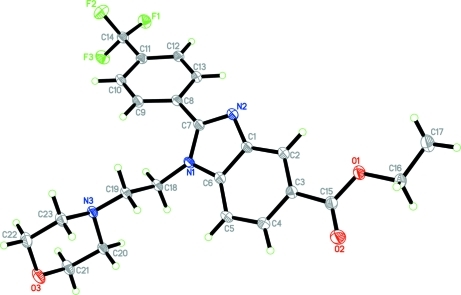
The asymmetric unit of (I), showing 30% probability displacement ellipsoids.

**Fig. 2.**
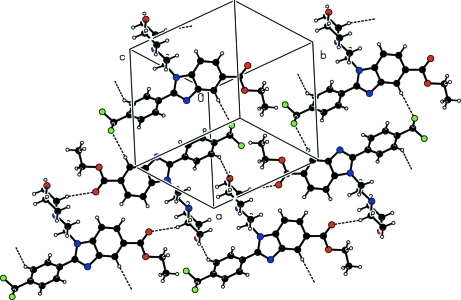
The crystal packing of the title compound (I).

## supplementary crystallographic information

### Comment

A wide variety of benzimidazole derivatives have been described for their chemotherapeutic importance (Boruah & Skibo, 1994). The synthesis of novel benzimidazole derivatives remains an important focus in medicinal research. Recent observations suggest that substituted benzimidazoles and heterocyclic, which are the structural isosters of nucleotides owing to their fused heterocyclic nuclei in their structures that allow them to interact easily with the biopolymers, possess potential activity with lower toxicities in the chemotherapeutic approach in man (Haugwitz, 1982; Hisano, 1982). Moreover, these fused heterocylces were distinctively studied for their antitumor, antiviral and antimicrobial activities as new nonnucleoside topoisomerase I poisons, human immunodeficiency virus-1 reverse transcriptase inhibitors and or potent DNA gyrase inhibitors (Hubschwerlen, 1992; Shi, 1996). In addition, benzimidazole derivatives have played a crucial role in the theoretical development of heterocyclic chemistry and are also used extensively in organic synthesis.

The molecular structure of the title compound, (I), is shown in Fig. 1. The benzimidazole (N1–N2/C1–C7) ring is approximately planar with maximum deviation of 0.028 (1) Å for atom C4.  The morpholine (N3/O3/C20–C23) ring adopts a chair conformation [Q = 0.5778 (12) Å, θ = 178.81 (12)°, φ = 128 (5)°; Cremer & Pople, 1975]. The central benzimidazole (N1–N2/C1–C7) ring makes dihedral angles of 35.66 (4)° and 75.45 (5)° with the attached phenyl (C8–C13) and the morpholine (N3/O3/C20–C23) rings, respectively.

In the crystal (Fig. 2), adjacent molecules are connected via intermolecular C2—H2A···F1, C10—H10A···O3 and C20—H20A···O2 (Table 1) hydrogen bonds to form a two-dimensional network.

### Experimental

Ethlyl-3-amino-4-(morpholinoethylamino) benzoate (0.01 mol) and sodium metabisulfite adduct of trifluromethyl benzaldehyde (0.01 mol) were dissolved in DMF. The reaction mixture was refluxed at 130°C for 4 h. After completion, the reaction mixture was diluted in ethyl acetate (20 ml) and washed with water (20 ml). The organic layer was collected, dried over Na_2_SO_4_ and the evaporated in vacuo to yield the product. The product was recrystallised from ethyl acetate to yield colourless blocks of (I).

### Refinement

All H atoms were positioned geometrically [C—H = 0.95–0.99 Å] and were refined using a riding model, with *U*_iso_(H) = 1.2*U*_eq_(C). A rotating group model was used for the methyl group.

### Crystal data

**Table:**

C_23_H_24_F_3_N_3_O_3_	*Z* = 2
*M**_r_* = 447.45	*F*(000) = 468
Triclinic, *P*1	*D*_x_ = 1.414 Mg m^−^^3^
Hall symbol: -P 1	Mo *K*α radiation, λ = 0.71073 Å
*a* = 10.1463 (2) Å	Cell parameters from 9996 reflections
*b* = 10.5595 (2) Å	θ = 2.4–30.1°
*c* = 11.5775 (2) Å	µ = 0.11 mm^−^^1^
α = 96.868 (1)°	*T* = 100 K
β = 109.638 (1)°	Block, colourless
γ = 110.833 (1)°	0.51 × 0.33 × 0.19 mm
*V* = 1050.83 (3) Å^3^	

### Data collection

**Table:**

Bruker SMART APEXII CCD diffractometer	6122 independent reflections
Radiation source: fine-focus sealed tube	5266 reflections with *I* > 2σ(*I*)
graphite	*R*_int_ = 0.024
φ and ω scans	θ_max_ = 30.2°, θ_min_ = 1.9°
Absorption correction: multi-scan (*SADABS*; Bruker, 2009)	*h* = −14→14
*T*_min_ = 0.945, *T*_max_ = 0.979	*k* = −14→14
22546 measured reflections	*l* = −16→15

### Refinement

**Table:**

Refinement on *F*^2^	Primary atom site location: structure-invariant direct methods
Least-squares matrix: full	Secondary atom site location: difference Fourier map
*R*[*F*^2^ > 2σ(*F*^2^)] = 0.038	Hydrogen site location: inferred from neighbouring sites
*wR*(*F*^2^) = 0.106	H-atom parameters constrained
*S* = 1.03	*w* = 1/[σ^2^(*F*_o_^2^) + (0.0561*P*)^2^ + 0.2843*P*] where *P* = (*F*_o_^2^ + 2*F*_c_^2^)/3
6122 reflections	(Δ/σ)_max_ = 0.001
290 parameters	Δρ_max_ = 0.43 e Å^−^^3^
0 restraints	Δρ_min_ = −0.26 e Å^−^^3^

### Special details

**Table:**

Experimental. The crystal was placed in the cold stream of an Oxford Cryosystems Cobra open-flow nitrogen cryostat (Cosier & Glazer, 1986) operating at 100.0 (1) K.
Geometry. All esds (except the esd in the dihedral angle between two l.s. planes) are estimated using the full covariance matrix. The cell esds are taken into account individually in the estimation of esds in distances, angles and torsion angles; correlations between esds in cell parameters are only used when they are defined by crystal symmetry. An approximate (isotropic) treatment of cell esds is used for estimating esds involving l.s. planes.
Refinement. Refinement of F^2^ against ALL reflections. The weighted R-factor wR and goodness of fit S are based on F^2^, conventional R-factors R are based on F, with F set to zero for negative F^2^. The threshold expression of F^2^ > 2sigma(F^2^) is used only for calculating R-factors(gt) etc. and is not relevant to the choice of reflections for refinement. R-factors based on F^2^ are statistically about twice as large as those based on F, and R- factors based on ALL data will be even larger.

### Fractional atomic coordinates and isotropic or equivalent isotropic displacement parameters (Å^2^)

**Table:**

	*x*	*y*	*z*	*U*_iso_*/*U*_eq_	
F1	0.57861 (8)	1.42374 (7)	0.30620 (7)	0.02777 (15)	
F2	0.46917 (8)	1.29209 (7)	0.11309 (6)	0.02950 (16)	
F3	0.71291 (8)	1.42604 (7)	0.19595 (7)	0.02843 (15)	
O1	0.69592 (9)	0.47558 (8)	0.74341 (7)	0.02040 (15)	
O2	0.85918 (10)	0.40173 (9)	0.69712 (8)	0.02932 (18)	
O3	1.19661 (9)	0.84859 (9)	0.01681 (7)	0.02600 (17)	
N1	0.83537 (9)	0.85327 (9)	0.38581 (8)	0.01618 (16)	
N2	0.65751 (9)	0.82663 (9)	0.46835 (8)	0.01700 (16)	
N3	0.98967 (9)	0.86116 (8)	0.13194 (7)	0.01548 (15)	
C1	0.73153 (11)	0.74099 (10)	0.50627 (9)	0.01619 (17)	
C2	0.71070 (11)	0.64983 (10)	0.58354 (9)	0.01726 (18)	
H2A	0.6343	0.6359	0.6167	0.021*	
C3	0.80658 (11)	0.58039 (10)	0.60970 (9)	0.01745 (18)	
C4	0.92137 (11)	0.60204 (10)	0.56165 (9)	0.01902 (18)	
H4A	0.9867	0.5550	0.5839	0.023*	
C5	0.94156 (11)	0.68952 (10)	0.48328 (9)	0.01836 (18)	
H5A	1.0176	0.7028	0.4498	0.022*	
C6	0.84348 (11)	0.75739 (10)	0.45626 (9)	0.01645 (17)	
C7	0.72211 (11)	0.89169 (10)	0.39757 (9)	0.01597 (17)	
C8	0.68579 (11)	1.00214 (10)	0.34664 (9)	0.01626 (17)	
C9	0.69019 (11)	1.02693 (10)	0.23121 (9)	0.01882 (18)	
H9A	0.7115	0.9671	0.1792	0.023*	
C10	0.66367 (11)	1.13828 (11)	0.19277 (9)	0.01912 (19)	
H10A	0.6688	1.1559	0.1155	0.023*	
C11	0.62940 (11)	1.22431 (10)	0.26810 (9)	0.01741 (18)	
C12	0.61869 (11)	1.19837 (10)	0.38023 (9)	0.01806 (18)	
H12A	0.5924	1.2558	0.4298	0.022*	
C13	0.64690 (11)	1.08740 (10)	0.41914 (9)	0.01761 (18)	
H13A	0.6397	1.0692	0.4957	0.021*	
C14	0.59851 (12)	1.34179 (11)	0.22216 (10)	0.02011 (19)	
C15	0.79184 (12)	0.47723 (11)	0.68685 (9)	0.01946 (19)	
C16	0.67213 (13)	0.37082 (11)	0.81448 (10)	0.0224 (2)	
H16A	0.7715	0.3881	0.8834	0.027*	
H16B	0.6290	0.2752	0.7573	0.027*	
C17	0.56154 (14)	0.38291 (12)	0.86957 (11)	0.0266 (2)	
H17A	0.5412	0.3120	0.9164	0.040*	
H17B	0.4645	0.3676	0.8006	0.040*	
H17C	0.6065	0.4771	0.9277	0.040*	
C18	0.94152 (11)	0.90747 (10)	0.32523 (9)	0.01693 (17)	
H18A	1.0476	0.9268	0.3839	0.020*	
H18B	0.9416	0.9972	0.3083	0.020*	
C19	0.89489 (11)	0.80215 (10)	0.20000 (9)	0.01663 (17)	
H19A	0.9059	0.7163	0.2182	0.020*	
H19B	0.7850	0.7747	0.1453	0.020*	
C20	1.15464 (11)	0.90256 (10)	0.20837 (9)	0.01811 (18)	
H20A	1.1691	0.8211	0.2350	0.022*	
H20B	1.1916	0.9786	0.2861	0.022*	
C21	1.24758 (12)	0.95334 (12)	0.13135 (10)	0.0243 (2)	
H21A	1.2383	1.0386	0.1099	0.029*	
H21B	1.3579	0.9794	0.1835	0.029*	
C22	1.03751 (13)	0.81211 (12)	−0.05885 (10)	0.0251 (2)	
H22A	1.0017	0.7401	−0.1390	0.030*	
H22B	1.0260	0.8964	−0.0811	0.030*	
C23	0.93906 (12)	0.75531 (11)	0.01281 (9)	0.02070 (19)	
H23A	0.8295	0.7302	−0.0411	0.025*	
H23B	0.9475	0.6691	0.0324	0.025*	

### Atomic displacement parameters (Å^2^)

**Table:**

	*U*^11^	*U*^22^	*U*^33^	*U*^12^	*U*^13^	*U*^23^
F1	0.0374 (4)	0.0265 (3)	0.0319 (3)	0.0204 (3)	0.0199 (3)	0.0113 (3)
F2	0.0268 (3)	0.0292 (3)	0.0280 (3)	0.0123 (3)	0.0044 (3)	0.0108 (3)
F3	0.0285 (3)	0.0250 (3)	0.0394 (4)	0.0103 (3)	0.0210 (3)	0.0170 (3)
O1	0.0242 (4)	0.0216 (3)	0.0227 (3)	0.0115 (3)	0.0144 (3)	0.0110 (3)
O2	0.0365 (5)	0.0326 (4)	0.0379 (4)	0.0238 (4)	0.0239 (4)	0.0197 (4)
O3	0.0221 (4)	0.0328 (4)	0.0221 (4)	0.0077 (3)	0.0141 (3)	0.0018 (3)
N1	0.0163 (4)	0.0184 (4)	0.0177 (4)	0.0081 (3)	0.0103 (3)	0.0056 (3)
N2	0.0174 (4)	0.0192 (4)	0.0174 (4)	0.0086 (3)	0.0095 (3)	0.0057 (3)
N3	0.0150 (4)	0.0176 (4)	0.0145 (3)	0.0055 (3)	0.0083 (3)	0.0043 (3)
C1	0.0162 (4)	0.0176 (4)	0.0162 (4)	0.0076 (3)	0.0082 (3)	0.0034 (3)
C2	0.0175 (4)	0.0195 (4)	0.0176 (4)	0.0082 (3)	0.0098 (3)	0.0056 (3)
C3	0.0190 (4)	0.0182 (4)	0.0164 (4)	0.0080 (3)	0.0085 (3)	0.0049 (3)
C4	0.0198 (4)	0.0210 (4)	0.0199 (4)	0.0109 (4)	0.0099 (4)	0.0053 (3)
C5	0.0173 (4)	0.0210 (4)	0.0202 (4)	0.0089 (4)	0.0109 (3)	0.0050 (3)
C6	0.0167 (4)	0.0170 (4)	0.0161 (4)	0.0064 (3)	0.0085 (3)	0.0034 (3)
C7	0.0159 (4)	0.0180 (4)	0.0158 (4)	0.0074 (3)	0.0085 (3)	0.0039 (3)
C8	0.0151 (4)	0.0176 (4)	0.0170 (4)	0.0064 (3)	0.0081 (3)	0.0046 (3)
C9	0.0213 (5)	0.0213 (4)	0.0181 (4)	0.0102 (4)	0.0116 (4)	0.0056 (3)
C10	0.0198 (4)	0.0231 (5)	0.0197 (4)	0.0098 (4)	0.0125 (4)	0.0081 (4)
C11	0.0157 (4)	0.0178 (4)	0.0197 (4)	0.0067 (3)	0.0085 (3)	0.0061 (3)
C12	0.0181 (4)	0.0206 (4)	0.0170 (4)	0.0093 (4)	0.0081 (3)	0.0036 (3)
C13	0.0182 (4)	0.0213 (4)	0.0159 (4)	0.0091 (4)	0.0091 (3)	0.0053 (3)
C14	0.0195 (5)	0.0208 (4)	0.0223 (4)	0.0084 (4)	0.0106 (4)	0.0075 (4)
C15	0.0207 (5)	0.0205 (4)	0.0184 (4)	0.0088 (4)	0.0090 (4)	0.0059 (3)
C16	0.0252 (5)	0.0226 (5)	0.0237 (5)	0.0107 (4)	0.0121 (4)	0.0124 (4)
C17	0.0309 (6)	0.0267 (5)	0.0272 (5)	0.0115 (4)	0.0170 (4)	0.0111 (4)
C18	0.0164 (4)	0.0181 (4)	0.0180 (4)	0.0056 (3)	0.0110 (3)	0.0043 (3)
C19	0.0153 (4)	0.0173 (4)	0.0178 (4)	0.0049 (3)	0.0100 (3)	0.0036 (3)
C20	0.0151 (4)	0.0216 (4)	0.0170 (4)	0.0056 (3)	0.0086 (3)	0.0040 (3)
C21	0.0198 (5)	0.0266 (5)	0.0224 (5)	0.0028 (4)	0.0131 (4)	0.0022 (4)
C22	0.0242 (5)	0.0318 (5)	0.0175 (4)	0.0080 (4)	0.0115 (4)	0.0040 (4)
C23	0.0190 (4)	0.0230 (5)	0.0165 (4)	0.0050 (4)	0.0088 (3)	0.0011 (3)

### Geometric parameters (Å, °)

**Table:**

F1—C14	1.3384 (12)	C9—H9A	0.9500
F2—C14	1.3528 (12)	C10—C11	1.3950 (13)
F3—C14	1.3407 (12)	C10—H10A	0.9500
O1—C15	1.3399 (12)	C11—C12	1.3895 (13)
O1—C16	1.4561 (12)	C11—C14	1.4975 (14)
O2—C15	1.2132 (13)	C12—C13	1.3915 (13)
O3—C21	1.4251 (13)	C12—H12A	0.9500
O3—C22	1.4311 (13)	C13—H13A	0.9500
N1—C6	1.3815 (12)	C16—C17	1.4986 (15)
N1—C7	1.3883 (12)	C16—H16A	0.9900
N1—C18	1.4646 (12)	C16—H16B	0.9900
N2—C7	1.3224 (12)	C17—H17A	0.9800
N2—C1	1.3896 (12)	C17—H17B	0.9800
N3—C19	1.4610 (12)	C17—H17C	0.9800
N3—C23	1.4704 (12)	C18—C19	1.5303 (13)
N3—C20	1.4722 (12)	C18—H18A	0.9900
C1—C2	1.4000 (13)	C18—H18B	0.9900
C1—C6	1.4077 (13)	C19—H19A	0.9900
C2—C3	1.3922 (13)	C19—H19B	0.9900
C2—H2A	0.9500	C20—C21	1.5139 (13)
C3—C4	1.4124 (13)	C20—H20A	0.9900
C3—C15	1.4878 (13)	C20—H20B	0.9900
C4—C5	1.3818 (14)	C21—H21A	0.9900
C4—H4A	0.9500	C21—H21B	0.9900
C5—C6	1.3977 (13)	C22—C23	1.5152 (14)
C5—H5A	0.9500	C22—H22A	0.9900
C7—C8	1.4724 (13)	C22—H22B	0.9900
C8—C13	1.4019 (13)	C23—H23A	0.9900
C8—C9	1.4042 (13)	C23—H23B	0.9900
C9—C10	1.3865 (14)		
			
C15—O1—C16	114.83 (8)	F2—C14—C11	111.39 (8)
C21—O3—C22	109.12 (8)	O2—C15—O1	123.35 (9)
C6—N1—C7	106.10 (8)	O2—C15—C3	123.50 (9)
C6—N1—C18	123.21 (8)	O1—C15—C3	113.15 (8)
C7—N1—C18	130.41 (8)	O1—C16—C17	107.55 (8)
C7—N2—C1	105.02 (8)	O1—C16—H16A	110.2
C19—N3—C23	109.01 (7)	C17—C16—H16A	110.2
C19—N3—C20	111.75 (7)	O1—C16—H16B	110.2
C23—N3—C20	108.99 (7)	C17—C16—H16B	110.2
N2—C1—C2	129.84 (9)	H16A—C16—H16B	108.5
N2—C1—C6	109.97 (8)	C16—C17—H17A	109.5
C2—C1—C6	120.18 (9)	C16—C17—H17B	109.5
C3—C2—C1	117.21 (9)	H17A—C17—H17B	109.5
C3—C2—H2A	121.4	C16—C17—H17C	109.5
C1—C2—H2A	121.4	H17A—C17—H17C	109.5
C2—C3—C4	121.52 (9)	H17B—C17—H17C	109.5
C2—C3—C15	122.16 (9)	N1—C18—C19	111.25 (8)
C4—C3—C15	116.30 (9)	N1—C18—H18A	109.4
C5—C4—C3	122.02 (9)	C19—C18—H18A	109.4
C5—C4—H4A	119.0	N1—C18—H18B	109.4
C3—C4—H4A	119.0	C19—C18—H18B	109.4
C4—C5—C6	116.02 (9)	H18A—C18—H18B	108.0
C4—C5—H5A	122.0	N3—C19—C18	111.69 (7)
C6—C5—H5A	122.0	N3—C19—H19A	109.3
N1—C6—C5	131.06 (9)	C18—C19—H19A	109.3
N1—C6—C1	105.89 (8)	N3—C19—H19B	109.3
C5—C6—C1	122.99 (9)	C18—C19—H19B	109.3
N2—C7—N1	113.01 (8)	H19A—C19—H19B	107.9
N2—C7—C8	122.72 (8)	N3—C20—C21	110.18 (8)
N1—C7—C8	124.07 (8)	N3—C20—H20A	109.6
C13—C8—C9	118.97 (9)	C21—C20—H20A	109.6
C13—C8—C7	117.53 (8)	N3—C20—H20B	109.6
C9—C8—C7	123.49 (8)	C21—C20—H20B	109.6
C10—C9—C8	120.37 (9)	H20A—C20—H20B	108.1
C10—C9—H9A	119.8	O3—C21—C20	111.87 (8)
C8—C9—H9A	119.8	O3—C21—H21A	109.2
C9—C10—C11	119.71 (9)	C20—C21—H21A	109.2
C9—C10—H10A	120.1	O3—C21—H21B	109.2
C11—C10—H10A	120.1	C20—C21—H21B	109.2
C12—C11—C10	120.83 (9)	H21A—C21—H21B	107.9
C12—C11—C14	121.00 (9)	O3—C22—C23	110.72 (8)
C10—C11—C14	118.13 (9)	O3—C22—H22A	109.5
C11—C12—C13	119.28 (9)	C23—C22—H22A	109.5
C11—C12—H12A	120.4	O3—C22—H22B	109.5
C13—C12—H12A	120.4	C23—C22—H22B	109.5
C12—C13—C8	120.76 (9)	H22A—C22—H22B	108.1
C12—C13—H13A	119.6	N3—C23—C22	110.37 (8)
C8—C13—H13A	119.6	N3—C23—H23A	109.6
F1—C14—F3	107.03 (8)	C22—C23—H23A	109.6
F1—C14—F2	106.57 (8)	N3—C23—H23B	109.6
F3—C14—F2	106.04 (8)	C22—C23—H23B	109.6
F1—C14—C11	112.96 (8)	H23A—C23—H23B	108.1
F3—C14—C11	112.41 (8)		
			
C7—N2—C1—C2	179.25 (10)	C9—C10—C11—C14	−178.85 (9)
C7—N2—C1—C6	0.28 (10)	C10—C11—C12—C13	1.81 (15)
N2—C1—C2—C3	−177.58 (9)	C14—C11—C12—C13	179.42 (9)
C6—C1—C2—C3	1.29 (14)	C11—C12—C13—C8	0.00 (15)
C1—C2—C3—C4	0.72 (14)	C9—C8—C13—C12	−2.40 (14)
C1—C2—C3—C15	−177.84 (9)	C7—C8—C13—C12	176.78 (9)
C2—C3—C4—C5	−2.01 (15)	C12—C11—C14—F1	7.10 (13)
C15—C3—C4—C5	176.62 (9)	C10—C11—C14—F1	−175.22 (9)
C3—C4—C5—C6	1.14 (14)	C12—C11—C14—F3	128.35 (10)
C7—N1—C6—C5	−176.49 (10)	C10—C11—C14—F3	−53.98 (12)
C18—N1—C6—C5	−1.98 (16)	C12—C11—C14—F2	−112.80 (10)
C7—N1—C6—C1	0.82 (10)	C10—C11—C14—F2	64.87 (12)
C18—N1—C6—C1	175.33 (8)	C16—O1—C15—O2	−2.71 (14)
C4—C5—C6—N1	177.86 (9)	C16—O1—C15—C3	176.96 (8)
C4—C5—C6—C1	0.94 (14)	C2—C3—C15—O2	169.88 (10)
N2—C1—C6—N1	−0.71 (10)	C4—C3—C15—O2	−8.74 (15)
C2—C1—C6—N1	−179.79 (8)	C2—C3—C15—O1	−9.78 (13)
N2—C1—C6—C5	176.88 (9)	C4—C3—C15—O1	171.59 (8)
C2—C1—C6—C5	−2.20 (14)	C15—O1—C16—C17	−179.41 (9)
C1—N2—C7—N1	0.27 (11)	C6—N1—C18—C19	79.90 (11)
C1—N2—C7—C8	−174.75 (8)	C7—N1—C18—C19	−107.04 (11)
C6—N1—C7—N2	−0.71 (11)	C23—N3—C19—C18	−179.88 (8)
C18—N1—C7—N2	−174.67 (9)	C20—N3—C19—C18	59.59 (10)
C6—N1—C7—C8	174.22 (8)	N1—C18—C19—N3	173.79 (8)
C18—N1—C7—C8	0.26 (15)	C19—N3—C20—C21	175.96 (8)
N2—C7—C8—C13	31.52 (13)	C23—N3—C20—C21	55.41 (10)
N1—C7—C8—C13	−142.93 (9)	C22—O3—C21—C20	59.43 (12)
N2—C7—C8—C9	−149.33 (10)	N3—C20—C21—O3	−58.04 (12)
N1—C7—C8—C9	36.21 (14)	C21—O3—C22—C23	−59.79 (11)
C13—C8—C9—C10	3.05 (15)	C19—N3—C23—C22	−178.76 (8)
C7—C8—C9—C10	−176.09 (9)	C20—N3—C23—C22	−56.55 (11)
C8—C9—C10—C11	−1.29 (15)	O3—C22—C23—N3	59.56 (12)
C9—C10—C11—C12	−1.17 (15)		

### Hydrogen-bond geometry (Å, °)

**Table:**

*D*—H···*A*	*D*—H	H···*A*	*D*···*A*	*D*—H···*A*
C2—H2A···F1^i^	0.95	2.51	3.4617 (15)	175
C10—H10A···O3^ii^	0.95	2.38	3.1889 (14)	143
C20—H20A···O2^iii^	0.99	2.52	3.4878 (14)	166

Symmetry codes: (i) −*x*+1, −*y*+2, −*z*+1; (ii) −*x*+2, −*y*+2, −*z*; (iii) −*x*+2, −*y*+1, −*z*+1.
